# Bacterial Community Dynamics in an Oyster Hatchery in Response to Probiotic Treatment

**DOI:** 10.3389/fmicb.2019.01060

**Published:** 2019-05-15

**Authors:** Rebecca J. Stevick, Saebom Sohn, Tejashree H. Modak, David R. Nelson, David C. Rowley, Karin Tammi, Roxanna Smolowitz, Kathryn Markey Lundgren, Anton F. Post, Marta Gómez-Chiarri

**Affiliations:** ^1^Graduate School of Oceanography, The University of Rhode Island, Narragansett, RI, United States; ^2^Department of Fisheries, Animal and Veterinary Sciences, The University of Rhode Island, Kingston, RI, United States; ^3^Department of Cell and Molecular Biology, The University of Rhode Island, Kingston, RI, United States; ^4^Department of Biomedical and Pharmaceutical Sciences, College of Pharmacy, The University of Rhode Island, Kingston, RI, United States; ^5^Feinstein School of Social and Natural Sciences, Roger Williams University, Bristol, RI, United States; ^6^Division of Research, Florida Atlantic University, Boca Raton, FL, United States

**Keywords:** microbiome, 16S rRNA sequencing analysis, oyster hatchery, probiotics, *Vibrio*, *Crassostrea virginica*, larvae

## Abstract

Larval oysters in hatcheries are susceptible to diseases caused by bacterial pathogens, including *Vibrio* spp. Previous studies have shown that daily addition of the probiotic *Bacillus pumilus* RI06-95 to water in rearing tanks increases larval survival when challenged with the pathogen *Vibrio coralliilyticus*. We propose that the presence of probiotics causes shifts in bacterial community structure in rearing tanks, leading to a net decrease in the relative abundance of potential pathogens. During three trials spanning the 2012–2015 hatchery seasons, larvae, tank biofilm, and rearing water samples were collected from control and probiotic-treated tanks in an oyster hatchery over a 12-day period after spawning. Samples were analyzed by 16S rRNA sequencing of the V4 or V6 regions followed by taxonomic classification, in order to determine bacterial community structures. There were significant differences in bacterial composition over time and between sample types, but no major effect of probiotics on the structure and diversity of bacterial communities (phylum level, Bray–Curtis *k* = 2, 95% confidence). Probiotic treatment, however, led to a higher relative percent abundance of *Oceanospirillales* and *Bacillus* spp. in water and oyster larvae. In the water, an increase in *Vibrio* spp. diversity in the absence of a net increase in relative read abundance suggests a likely decrease in the abundance of specific pathogenic *Vibrio* spp., and therefore lower chances of a disease outbreak. Co-occurrence network analysis also suggests that probiotic treatment had a systemic effect on targeted members of the bacterial community, leading to a net decrease in potentially pathogenic species.

## Introduction

Diseases caused by bacterial pathogens result in losses in aquaculture and wild populations of commercially important shellfish and finfish (Lafferty et al., 2015; Groner et al., 2016; Pérez-Sánchez et al., 2018). World aquaculture production is valued at $243.5 billion USD, and disease is a primary limiting factor on its growth and economic worth (Stentiford et al., 2012; FAO, 2018). Larval oysters are especially susceptible to disease, often by etiological agents from the genus *Vibrio* (Beaz-Hidalgo et al., 2010a; Richards et al., 2015; Le Roux et al., 2016; Dubert et al., 2017; King et al., 2018). Pathogenic *Vibrio* spp. are naturally occurring microbes in coastal waters, which makes them difficult to avoid. In an effort to maintain a healthy environment, hatcheries work toward optimum water quality by controlling larval culture density and the use of water treatment systems (Mckindsey et al., 2007; Pérez-Sánchez et al., 2018).

An alternative method for the management of disease in aquaculture involves the use of probiotics, microorganisms that provide health benefits to the host, including protection against bacterial pathogens. Probiotics exert their beneficial effects through a variety of mechanisms, including direct pathogen inhibition, competition for nutrients, secretion of antibacterial substances, and improvement of water quality (Kesarcodi-Watson et al., 2008, 2012; Prado et al., 2010). Previous studies have shown that treatment of larval oysters in the laboratory or the hatchery with the probiotic bacterium *Bacillus pumilus* RI06-95 significantly increases their survival when challenged with the pathogen *Vibrio coralliilyticus* (Karim et al., 2013; Sohn et al., 2016a). Additionally, administration of this probiotic in a hatchery setting results in reductions in total *Vibrio* abundance in tank water and surfaces, compared to the control tanks (Sohn et al., 2016b).

However, there is a lack of knowledge regarding the effects of probiotics on the systems in which they are used. There are concerns about using probiotic bacteria to combat disease in open aquaculture systems, as they will eventually disperse into the environment and may thus affect bacterial diversity in these systems (Newaj-Fyzul et al., 2014). Improper selection of probiotics may result in bacterial dysbiosis, which could ultimately impact host health (Verschuere et al., 2000). As filter feeders that process large volumes of seawater daily, bivalves are especially susceptible to changes in bacterial community composition in the water (Burge et al., 2016). Moreover, bacteria both contribute to and serve as indicators of oyster health and function of the microbial community (Le Roux et al., 2016) and likely mediate the effects of probiotics on the host. Therefore, it is important to assess the effects of probiotics not only on the health and protection of the host, but also on the bacterial communities in the systems in which oysters are grown.

Previous studies of microbiomes in adult oysters have shown differences in microbiota according to tissue type, geographic location, season, and environmental conditions (King et al., 2012; Chauhan et al., 2014; Lokmer and Wegner, 2015; Lokmer et al., 2016b; Pierce et al., 2016; Pierce and Ward, 2018). Additionally, the oyster microbiomes are distinct from those of the surrounding water and are often dominated by *Proteobacteria, Cyanobacteria*, and *Firmicutes* (Lokmer et al., 2016a). Three independent microbiome studies of larval cultures of the Pacific oyster, *Crassostrea gigas* found that, even though the microbiome in the rearing water changes throughout the year, there is little effect from direct manipulation of rearing conditions themselves, including salinity and temperature (Powell et al., 2013; Trabal Fernández et al., 2014; Asmani et al., 2016). Microbiome studies of juvenile Kumamoto oysters treated with *Streptomyces* N7 and RL8 showed an increase in species diversity and changes in the relative abundances of taxa, compared to control oysters (García Bernal et al., 2017). However, the effect of probiotics on bacterial communities in an oyster hatchery has not yet been determined.

In this study, we analyzed the structure and diversity of bacterial communities in larval oysters, their rearing water, and in tank biofilms over a 12-day period following treatment with the probiotic *B. pumilus* RI06-95. We hypothesized that probiotic treatment has a cascading effect on the bacterial community structure that alters the microbiomes of the rearing water, tank biofilms, and larvae, leading to a net decrease in potentially pathogenic species.

## Materials and Methods

### Bacterial Strain and Culture Conditions

The probiotic strain *B. pumilus* RI06-95, previously isolated from a marine sponge from the Pettaquamscutt River in Rhode Island (Karim et al., 2013), was cultured in yeast peptone with 3% salt (mYP30) media [5 g L^-1^ of peptone, 1 g L^-1^ of yeast extract, and 30 g L^-1^ of ocean salt (Red Sea Salt, Ohio, United States)] at 28°C with shaking at 170 rpm. The bacterial cell concentration was estimated by OD_550_ measurements using a spectrophotometer (Synergy HT, BioTek, United States) and confirmed using serial dilution and spot plating on mYP30 agar plates to determine colony forming units (CFU).

### Experimental Design and Sample Collection

Samples for microbiome analysis were collected during 3 hatchery trials performed at the Blount Shellfish Hatchery at Roger William University (Bristol, RI, United States) (Table 1). Eastern oysters (*Crassostrea virginica*) were spawned following standard procedures (Helm and Bourne, 2004). Spawning day is referred to as Day 0 throughout the manuscript. Larvae (1-day old) were distributed and maintained in static conditions in triplicate 120 L conical tanks for each treatment containing filtered and UV sterilized seawater at 21 – 23 °C and a salinity of 28 psu. Tanks were randomly assigned to treatments including no probiotics (control) and probiotic treatment with probiotic *B. pumilus* RI06-95. The probiotic was administered daily at 10^4^ CFU/mL, regardless of the length of the trial, to treatment tanks after being mixed with the microalgal feed. The microalgae strains used for feeding included *Chaetoceros muelleri* (CCMP1316), *Isochrysis galbana* (CCMP1323), *Tisochrysis lutea* (CCMP1324), *Pavlova lutheri* (CCMP1325), *Tetraselmis* sp. (CCMP892), and *Thalassiosira weissflogii* (CCMP1336). Experimental tanks were drained every other day to perform larval counts and grading. Tanks were washed thoroughly with a diluted bleach solution, rinsed, and replenished with filtered and UV-treated water prior to restocking the larvae. Sampling timepoints and trial lengths varied according to the hatchery-scheduled drain down days, so that extensive larval counts would coincide with sampling days.

**Table 1:** Summary of probiotic trial information and sequencing data.

	Trial 1	Trial 2	Trial 3
Sample types	Water, Swabs, Oysters	Water, Swabs, Oysters	Water
Sampling days (0 = spawn)	W:1,12 / OS:5,12	W:1,9 / OS:6,9	W: 5,8,12
Volume water filtered	410–750 mL	7–10 mL	1300–1700 mL
Trial dates	July 11–23, 2012	Jan 9–18, 2013	June 3–15, 2016
Bacterial reads from 12 water samples	1.3 million	1.8 million	5.7 million
Methods	Mo Bio extraction MiSeq, 2x250 PE	Mo Bio extraction MiSeq, 2x250 PE	Puregene extraction HiSeq, 2x100 PE
16S region	V4 (515F/806R)	V4 (515F/806R)	V6 (967F/1064R)

*W, water; S, swab; O, oysters.*

Rearing water (volumes in Table 1) was collected from each of the triplicate tanks during drain-down and filtered through a 0.22 μm Sterivex filter (Millipore, Milford, MA, United States). The Sterivex filters were immediately frozen and stored at -80°C until DNA extraction. Biofilm swab samples were collected from the surface inside of each tank after drain-down of the water by swabbing a line of approximately 144 cm in length with sterile cotton swabs. The cotton tips of the swabs were stored in RNAlater (Ambion, Inc., Foster City, CA, United States). Oyster larvae were collected on a 55 μm sieve after drain-down of tank water and resuspended in 5 L of seawater. 10 mL of oyster larvae (from each tank, about 150 – 1500 larvae) were then placed into a sterile tube. In the laboratory, oyster larvae were collected on a 40 μm nylon membrane and rinsed with filtered sterile seawater (FSSW) to reduce loosely attached environmental bacteria. Swab and larvae samples were flash frozen in liquid nitrogen and stored at -80°C until DNA extraction. Extracts from swab, larvae, and water samples were cultured on selective media to perform culturable *Vibrio* counts following methods in Sohn et al. (2016b). All sample types were collected during Trials 1 and 2, but only water samples were collected during Trial 3 (Table 1). In Trial 3, water (1 – 2 L) was also collected from the inflow (water piped directly from the environment) and outflow (water collected after filtration and UV-treatment prior to reaching the hatchery tanks) and processed as described above for tank water.

### DNA Extraction, Amplification, and Sequencing

Total DNA from water samples was extracted from the filters using the PowerWater Sterivex DNA Isolation Kit (Mo Bio Laboratories, United States) according to manufacturer recommendations (Trials 1 and 2) or Gentra Puregene Reagents (Qiagen, Hilden, Germany) with an added proteinase K-lytic enzyme digestion step (Sinigalliano et al., 2007; Trial 3). In addition, total bacterial DNA from the tank biofilm swabs and oyster larvae were extracted using the PowerSoil DNA Isolation Kit (Mo Bio) with slight modifications detailed below. In brief, frozen pooled oyster larvae were ground in a mortar with a sterile pestle and then placed into bead tubes for extraction (Qiagen). The RNAlater samples containing the cotton tops of the swabs were placed directly into bead tubes. Bead tubes were incubated at 65°C for 10 min and then shaken horizontally at maximum speed for 10 min using the Mo Bio vortex adaptor. Following extraction, DNA concentration was quantified with both a Nanodrop 2000 instrument and a Qubit Fluorometer (Thermo Fisher Scientific, Wilmington, DE, United States). The performance and quality of DNA extractions was comparable between trials and sample types.

16S rRNA gene amplicon analysis was performed using 515F/806R primers to amplify the V4 region (Trials 1 and 2) or 967F/1064R primers to amplify the V6 region (Trial 3). The V4 region was used in Trials 1 and 2 for better taxonomic resolution of all sample types and the V6 region was used in Trial 3 for independent confirmation with greater sequencing depth. A two-step PCR reaction following Illumina’s 16S Metagenomic Sequencing Library Preparation Protocol was performed on the samples from Trials 1 and 2 (Illumina Inc., San Diego, CA, United States). The PCR products were then analyzed with 250 bp paired-end sequencing to obtain fully overlapping reads on an Illumina MiSeq at the Genomics and Sequencing Center at the University of Rhode Island. The samples from Trial 3 were prepared with a 2-step fusion primer PCR amplification according to the protocols from the Keck Sequencing Center at the Marine Biological Laboratory (MBL^1^). Paired-end sequencing was performed at the MBL on an Illumina HiSeq 2500 to generate 100 bp paired-end reads with full overlap of the V6 region.

### Processing of Sequencing Data

Sequences from Trials 1 and 2 were demultiplexed using FastQC v0.11.4 (Andrews, 2010), then merged and trimmed using Trimmomatic v0.32 (Bolger et al., 2014). All sequences shorter than 200 bp were removed from the dataset. Sequences from Trial 3 were demultiplexed and quality filtered following standard protocols at the MBL Bay Paul Center that remove reads where forward and reverse sequences do not match perfectly (Eren et al., 2013b). All sequences were uploaded to VAMPS (Visualization and Analysis of Microbial Population Structure) and classified directly using the GAST pipeline with the SILVA database, in order to compare between the three trials (Huse et al., 2014). The taxonomy data from each trial were separately normalized to the total reads of each sample to provide relative abundance of each taxa in percentage, and then exported as a matrix or BIOM file for analysis in R (Version 3.3.1). *Vibrio* spp. sequences in water samples from Trial 3 were processed through the oligotyping pipeline described in Eren et al. (2013a) as implemented in VAMPS, and annotated using SILVA.

### Statistical and Network Analysis

All descriptive and statistical analyses were performed in the R statistical computing environment with the *vegan* and *phyloseq* packages (Dixon, 2003; McMurdie and Holmes, 2013). Simpson’s diversity values were calculated for each sample at the order level using the *vegan* package Version 2.4-1 and analyzed using the non-parametric Kruskal–Wallis rank sum test in R. Non-metric dimensional analysis (NMDS) was used to determine the influence of time, probiotic treatment, or sample type on the bacterial community composition, based on methods by Torondel et al. (2016) and implemented using *vegan*. The Bray-Curtis dissimilarity metric was calculated with *k* = 2 for max 50 iterations and 95% confidence intervals (standard deviation) were plotted. Statistical testing of the beta-diversity was done using the adonis2 test implemented in *vegan* (method = ”bray”, *k* = 2) (Mcardle and Anderson, 2010; Warton et al., 2012). Additionally, relative percent abundances of specific taxa were extracted and plotted according to treatment and time, and analyzed using the Kruskal–Wallis test in R.

A co-occurrence network was generated with normalized taxa counts at the Order level from water samples in Trial 3 (*n* = 18) to determine hypothetical relationships resulting from each treatment. The make_network() command from the *phyloseq* package was used with the Bray–Curtis dissimilarity metric, max distance = 0.5. The mean resulting relationship table including 123 taxa (nodes) and 670 relationships (edges) was exported to Cytoscape Version 3.6.0 for visualization and analysis (Shannon et al., 2003). Nodes were assigned continuous size attributes based on the number of normalized reads in all samples per taxa (2 to 2,720,021), and discrete shape and continuous color according to whether the taxa were more abundant in the control or probiotic-treated samples (0 to 3.6 times).

## Results

### Bacterial Structure and Diversity Over Time

**FIGURE 1:**
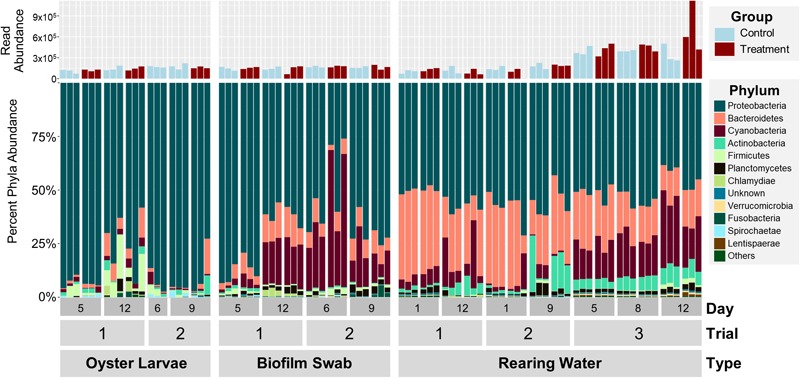
Percent abundances of the 12 most abundant phyla in oyster larvae, biofilm swab, and rearing water samples from all 3 trials based on 16S rRNA amplicon sequencing data (bottom). The total abundance of quality filtered sequencing reads is shown in the bar graph (top). The 12 dominant phyla include *Actinobacteria, Bacteroidetes, Cyanobacteria, Deferribacteres, Firmicutes, Fusobacteria, Lentisphaerae, Planctomycetes, Proteobacteria, Spirochaetae, Verrucomicrobia*, and Unknown. Note: there are no treated oyster larvae samples from Trial 2, Day 6.

In order to determine the effect of probiotics on the microbial community dynamics in an oyster hatchery, we needed to first characterize bacterial structure and diversity in different environmental niches within the hatchery (water, tank surfaces, and larvae) over time. A total of 18,103,647 quality-controlled 16S rRNA gene amplicon sequences were analyzed from 42 rearing water samples, 24 tank biofilm swabs, and 21 pooled larvae samples from three hatchery trials. There was an average of 208,087 reads for each of the 87 samples, ranging between 961 and 1,117,380 depending on the sequencing method and sample type (Figure 1, top). Direct taxonomical classification resulted in the detection of a total of 168 Orders across 29 Phyla in all samples. Overall, bacterial communities for each trial and sample type shared many of the most dominant phyla, although differences in relative abundance were seen between trials, timepoints, and sample types (Figure 1, bottom). The most dominant phyla in the water community, averaged from all samples, were *Proteobacteria* (53 ± 6%), *Bacteroidetes* (26 ± 10%), *Cyanobacteria* (12 ± 10%), *Actinobacteria* (5 ± 5%), and *Planctomycetes* (2 ± 1%) (Figure 1, bottom right). The larval samples were dominated by *Proteobacteria* (87 ± 12%) and the swab samples by *Proteobacteria* (68 ± 17%), *Cyanobacteria* (19 ± 16%), and *Bacteroidetes* (8 ± 4%) (Figure 1, bottom left). Percent abundance of *Cyanobacteria* was significantly higher in swab than in water samples (*p* < 0.001, Supplementary Table S1). Larval and swab samples showed a significantly higher proportion of *Proteobacteria*, and lower percent abundance of *Bacteroidetes*, as compared to water samples (*p* < 0.001, Supplementary Table S1). No significant effect of probiotic treatment was observed on the relative abundance of dominant phyla (*p* > 0.38).

**FIGURE 2:**
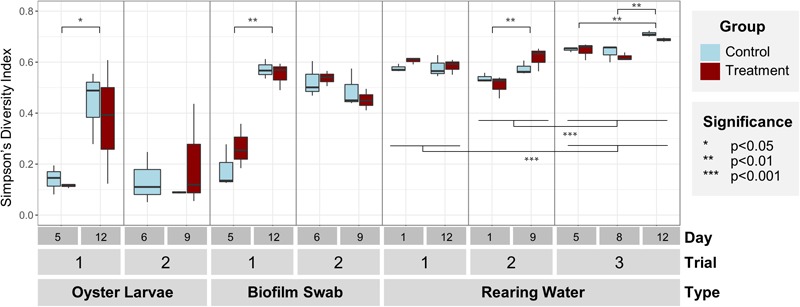
Simpson’s index of diversity of bacterial communities by sample (larvae, swab, and water) and trial (*n* = 3 tanks). No significant differences in diversity were found between control (light blue) and treatment (dark red) within each sample type and trial. Bacterial community diversity significantly increased over time in larvae and swab samples from Trial 1, and water samples from Trials 2 and 3. Diversity in water was significantly higher in Trial 3 than Trials 1 and 2. Note: there are no treated oyster larvae samples from Trial 2, Day 6.

Overall, the bacterial communities in rearing water were significantly more diverse than the communities in oyster larvae and tank biofilm swab samples (Simpson’s Diversity Index, *p* < 0.001, Figure 2 and Supplementary Table S2), reflecting an enrichment in specific community members in larvae and tank surfaces from the more diverse rearing water community (Figure 1). Simpson’s Diversity Index indicated significantly higher diversity in rearing water samples from Trial 3 (0.66 ± 0.04), than from Trials 1 (0.59 ± 0.3) and 2 (0.53 ± 0.5; *p* < 0.001, Figure 2, Supplementary Table S2), most probably due to the greater sequencing depth and different target 16S variable region in Trial 3 (Supplementary Figure S1), but potentially also due to seasonal and yearly differences in bacterial composition of the rearing water source (Table 1). There was high variability among replicate samples from each timepoint and treatment, especially in oyster larvae samples (Figure 2 and Supplementary Figure S2). Significant increases in bacterial diversity over time were detected in the oyster larvae and biofilm swabs in Trial 1 (*p* < 0.01, Supplementary Table S3), and in the rearing water in Trials 2 and 3 (*p* < 0.01, Figure 2 and Supplementary Tables S4, S5). No significant differences in Simpson’s Diversity Index were detected between control and treated samples at any timepoints for any of the sample types (*p* = 0.52).

**FIGURE 3:**
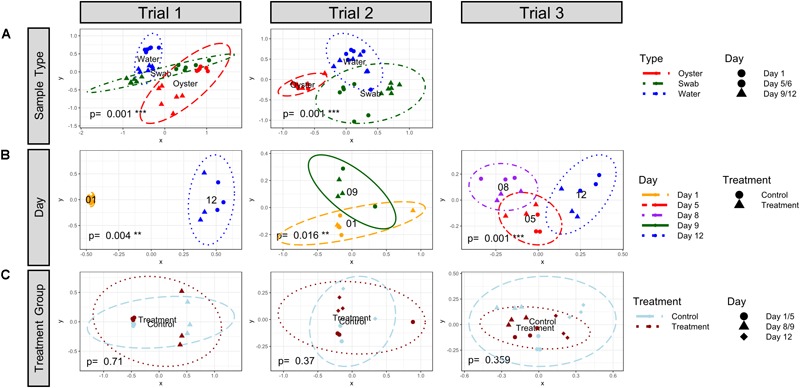
NMDS plot visualization of Bray-Curtis beta-diversity (*k* = 2) at the Order level by **(A)** sample type, **(B)** sampling day, and **(C)** treatment. The ellipse lines show the 95% confidence interval (standard deviation). *p*-values indicate significance of grouping with adonis2 Permutational Multivariate Analysis of Variance Using Distance Matrices test. **(A)** The different types of samples are indicated by colors (Oyster, dashed red; Swab, dashdot green; and Water, dotted blue) and the days are indicated by symbols (Timepoint 1, circle; Timepoint 2, triangle). The water and oyster communities were significantly distinct from each other in both trials. **(B)** The sampling timepoints are indicated by colors (1, longdash yellow; 5, shortdash red; 8, dashdot purple; 9, solid green; and 12, dotted blue) and the treatment group is indicated by symbols (control, circle; probiotic treatment, triangle). The water community was significantly different between timepoints. **(C)** The treatment group is indicated by colors (control, light blue dashed; probiotic treatment, dark red dotted) and sampling timepoints are indicated by symbols. No significant differences in community structure in water from control and probiotic-treated tanks was detected when samples from all timepoints were analyzed together.

The bacterial community structures of the water and oyster larvae samples were significantly different (Bray–Curtis, *k* = 2, 95% confidence, adonis2 *p* = 0.001) in both Trial 1 and Trial 2 (Figure 3A and Supplementary Table S6). The community structure of microbiomes in tank biofilms (swab samples) was not significantly different from the structure of either the water or oyster larvae samples, suggesting an intermediate microbiome stage. Bacterial communities in the rearing water were significantly different between sampling timepoints (Bray–Curtis, *k* = 2, 95% confidence, adonis2 *p* < 0.02) in all three trials (Figure 3B and Supplementary Table S6). Moreover, the bacterial community in samples of inflow and outflow seawater, which were collected on Days 5, 8, and 12 during Trial 3, was distinct from that of the water in rearing tanks (Supplementary Figure S3, adonis2 *p* = 0.001, and Supplementary Table S6). These results suggest that hatchery tanks containing oyster larvae have dynamically developing microbiomes, despite the fact that they are all receiving the same inflow seawater. There was no significant effect of treatment on the beta-diversity in water samples from all timepoints (Figure 3C and Supplementary Table S6).

### Effects of the Probiotic on the Selected Members of the Bacterial Community

**FIGURE 4:**
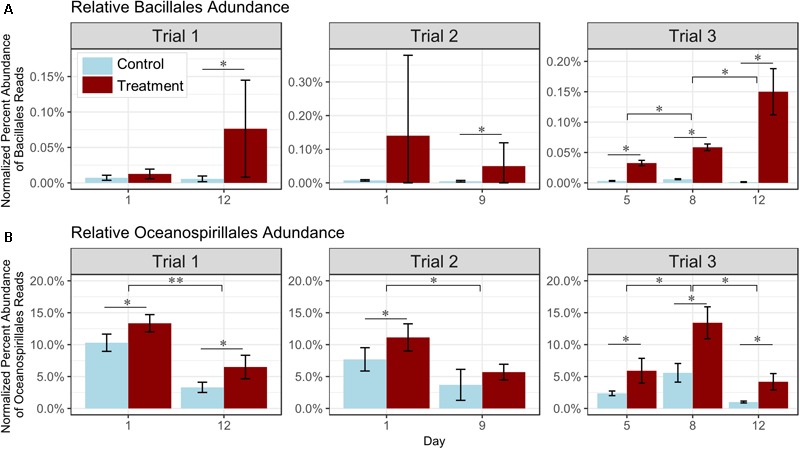
Effect of probiotic treatment on relative percent read abundance of **(A)** *Bacillales* and **(B)** *Oceanospirillales* in water. Number of reads in treated (dark red) and control (light blue) samples (*n* = 3 tanks per treatment) are represented for each sampling day and trial. **(A)** *Bacillales* was relatively significantly higher in the treated than the control water after 5 days of treatment, and **(B)** *Oceanospirillales* were consistently more abundant in probiotic-treated tank rearing water, and decreased with time. ^∗^*p* < 0.05, ^∗∗^*p* < 0.01, ^∗∗∗^*p* < 0.001.

Although control and probiotic-treated tanks showed no significant differences in diversity and structure of bacterial communities overall (Figure 3C), significant differences in the relative read abundance of several specific taxa were detected. In all trials, *Bacillales* reads in the probiotic-treated water samples increased through time, and were significantly more abundant in samples from treated tanks than in the control tanks by the final sampling day in all trials (*p* < 0.05, Figure 4A and Supplementary Table S7). These consistent results suggest that the relative increase in reads corresponded to the added probiotic. The relative percent of *Oceanospirillales* reads was also significantly higher by 20–34% at all but one timepoint in probiotic-treated rearing water as compared to control water in all trials (*p* < 0.05, Figure 4B and Supplementary Table S8). The relative percent abundance of *Oceanospirillales* reads in the water significantly decreased over time by 41–62% (depending on the trial; *p* < 0.05, Figure 4B and Supplementary Table S8). No significant changes in relative percent read abundance of these two selected members of the bacterial community were detected in larval oysters or swabs, but percent abundance was low in these sample types (Trials 1 and 2; not shown).

**FIGURE 5:**
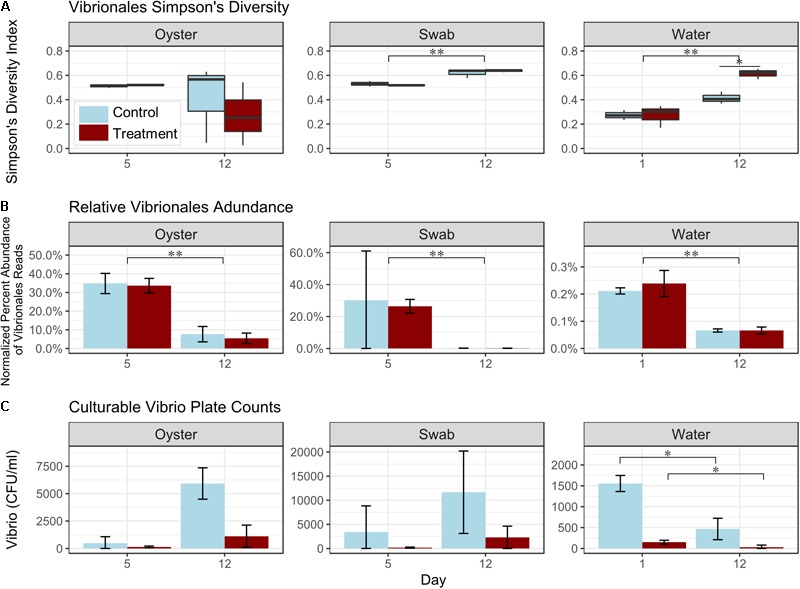
Effect of treatment, time, and sample type on Simpson’s Index of Diversity for *Vibrionales* (**A**, boxplots), total *Vibrionales* relative percent read abundance (**B**, bar graph), and culturable *Vibrio* plate counts (**C**, bar graph). Representative data from Trial 1 (*n* = 3 tanks per treatment). Note different scales for **(B)** and **(C)**. ^∗^*p* < 0.05, ^∗∗^*p* < 0.01, ^∗∗∗^*p* < 0.001.

*Vibrio* is a taxon that comprises a significant number of larval oyster pathogens, therefore we evaluated the effect of probiotic treatment on changes in *Vibrio* spp. diversity, relative abundance, and culturable colonies on selective media, over time during each of the hatchery trials (Figure 5 and Supplementary Figures S4, S5). Probiotic treatment led to a significant increase in *Vibrio* diversity (as measured using the Simpson’s Index of Diversity) in water samples collected on Day 12 in Trial 1 (*p* < 0.05; Figure 5A and Supplementary Table S9). No significant differences in relative percent abundance of *Vibrio* spp. between control and probiotic-treated tanks were detected for any of the sample types (Figure 5B and Supplementary Table S10). Colony counts of culturable *Vibrios*, however, were significantly lower in probiotic-treated tanks, relative to control tanks (*p* < 0.05, Figure 5C and Supplementary Table S11). When considering the effect of sample type, *Vibrio* relative abundance was significantly lower in water samples than in swabs or oysters (all timepoints) and in swabs than in oysters (*p* < 0.05, Figure 5B and Supplementary Table S10). When considering data from all timepoints together, the diversity of *Vibrio* spp. as detected using 16S rRNA gene sequencing was significantly higher in swab and oyster samples than in water samples (*p* < 0.05, Figure 5A and Supplementary Table S9). An evaluation of the effect of time on *Vibrio* relative abundance and diversity showed a significant increase in the diversity of *Vibrio* spp. in swab and water samples (Trial 1, *p* < 0.005, Figure 5A and Supplementary Table S9), and a significant decrease in relative abundance in all sample types (Trial 1, *p* < 0.005, Figure 5B and Supplementary Table S10). This decrease in abundance was further seen in colony counts of culturable *Vibrios* in the water samples (Trial 1, *p* < 0.05, Figure 5C and Supplementary Table S11).

**FIGURE 6:**
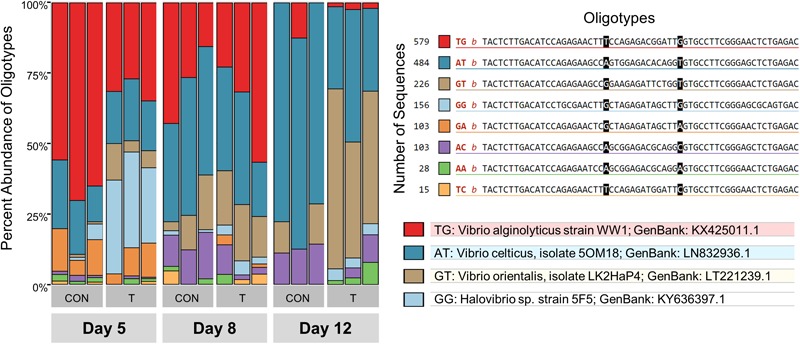
*Vibrio* spp. oligotypes in Control (CON) and Treatment (T) water samples on Days 5, 8, and 12 from Trial 3. These 8 oligotypes were generated from changes in positions 23 and 37 in a total of 1727 sequences, represented with the 2 letter abbreviations in the legend. The taxonomy of the 4 most abundant oligotypes is shown. *Vibrio* oligotypes showed differences in succession of species over time between control and treatment rearing water.

Since the V6 region of the 16S rRNA gene was deeply sequenced in Trial 3, we were able to perform an oligotyping analysis – a method that detects genetic variants within a taxon – of the 1,727 *Vibrio* reads in the 18 water samples. Changes in the overall composition of the *Vibrio* community over time and by treatment were observed by oligotyping (Figure 6). On Day 5, while the *Vibrio* community in control tanks was dominated by an oligotype most closely related to *V. alginolyticus* WW1 (64 ± 6%), probiotic tanks showed a mix of *V. alginolyticus* WW1 (31 ± 3%) and *Halovibrio* sp. 5F5 (31 ± 3%). By Day 12, the *Vibrio* composition in water in control tanks was dominated by *V. celticus* 5OM18 (75 ± 3%), while a mix of *V. orientalis* LK2HaP4 (51 ± 10%), and *V. celticus* 5OM18 (35 ± 8%) was detected in probiotic tanks.

### Bacterial Relationships With Co-occurrence Analysis

**FIGURE 7:**
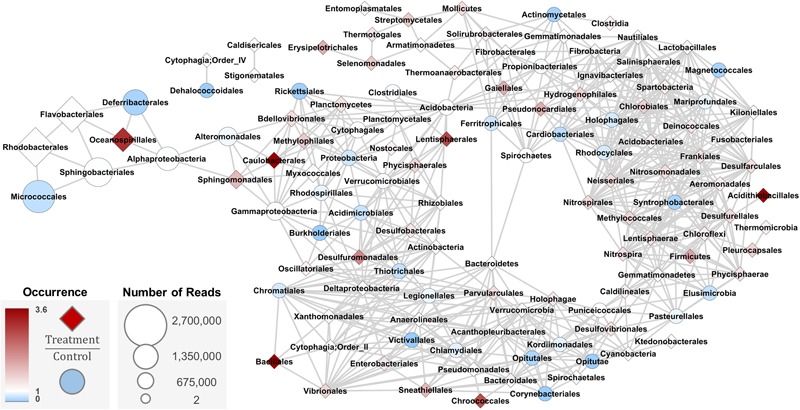
Co-occurrence network analysis based on Bray-Curtis dissimilarity metric (max distance = 0.5, Order level) for water samples from Trial 3 (*n* = 3 tanks per treatment and day, total of 18). Taxa that change in the same way share an edge; nodes that have edges occur in the same proportions and in the same samples. Darker blue circle nodes indicate taxa that occur in the Control significantly more than Treated water samples. White nodes have equal occurrence in treated and control water samples. Darker red diamond nodes indicated taxa that occur in the Treated significantly more than Control water samples.

A co-occurrence analysis of members of the bacterial community (Figure 7) in the 18 water samples from Trial 3 was performed to illustrate: (a) how abundance of each Order changed relative to others (edge connections); (b) which Orders were relatively most abundant in the system (node size); and (c) how probiotic treatment affected their relative abundances (node color and shape). The most abundant taxa (*Rhodobacterales, Micrococcales, Sphingobacteriales, Flavobacteriales, Deferribacterales*, and *Oceanospirillales*) changed in similar fashion, but had different occurrence ratios between control and treatment samples. Orders that were more abundant in the treatment samples than in control samples included *Oceanospirillales, Caulobacterales, Lentispherales, Acidithiobacillales, Chrococcales*, and *Bacillales*. These nodes were scattered throughout the network and did not share direct edges, but were within 3–5 edges of each other.

*Bacillales*, the Order to which the probiotic used in these experiments belongs, was shown to be most directly associated in the network with four other Orders that changed in relative abundance between control and treatment samples: *Chromatiales, Xanthomonadales, Cytophagia* Order II, and *Vibrionales*. This direct connection between *Bacillales* and *Vibrionales* in the network indicated that the probiotic may have directly affected members of *Vibrionales*. *Oceanospirillales* was placed in the network 5 edges away from *Bacillales*, sharing an edge with the treatment-abundant *Flavobacteriales*, a common environmental bacteria taxon. This network suggests that the probiotic did not directly alter the overall bacterial community in the rearing water in an oyster hatchery, but targeted specific members of the community.

## Discussion

A better understanding of bacterial community dynamics in aquaculture systems is critical for optimizing disease management strategies such as probiotic treatment. This study characterized: (a) changes in microbial communities in an oyster hatchery through the rearing process; and (b) the effect of probiotic treatment on those communities. To our knowledge, this is the first study to characterize the effects of probiotics on microbiomes in a bivalve hatchery. Despite the high spatial (by sample type and replicate tank) and temporal variability in bacterial composition at the hatchery detected in this research, results support the hypothesis that probiotic treatment leads to shifts in the microbial community in the hatchery from a state promoting the growth of potential pathogens to one that inhibits it.

Our results showed high variability in bacterial composition between replicate samples within trials and between trials, especially among the bacterial communities of oyster larvae. Variability between the 3 trials, conducted in July, January, and June in different years, is consistent with natural seasonal variation in microbial communities in Narragansett Bay (Staroscik and Smith, 2004). High variability in microbial communities in oysters from a single location is consistent with past studies, and is most probably driven by genetic and environmental effects on host-microbe interactions (King et al., 2012; Wegner et al., 2013). Moreover, variability between replicates (tanks within the hatchery) and between trials, may have been due to inevitable variance in husbandry and handling techniques at the hatchery (Elston et al., 1981, 2008).

Despite the high variability observed in these trials, our study observed clear differences in diversity and bacterial community structure between the rearing water, the biofilms on tank surfaces (swabs), and the oyster larvae. In particular, oyster larvae microbiomes were a subset of taxa present in the water and in biofilms, including *Firmicutes* and *Proteobacteria*, while tank biofilms showed a diversity and composition state that was intermediate between water and larvae. Lower diversity indices in the larvae and tank biofilms (swabs) than the water indicates niche selection of larval and biofilm colonizers, particularly *Cyanobacteria* in tank biofilms and *Proteobacteria* in oyster larvae. The dominance of *Proteobacteria* in the system, the most abundant phylum in all samples (up to 87% in larvae), is consistent with previous studies where it was shown to make up the largest and most diverse phylum in oyster-associated microbiota (Hernández-Zárate and Olmos-Soto, 2006; Trabal Fernández et al., 2014; Dittmann et al., 2018). Bacteria are an essential component of aquaculture nutrition, as a source of both nutrients and growth factors for the microalgae, and as food for the larvae (Kamiyama, 2004; Natrah et al., 2014; Nevejan et al., 2016). Factors such as size, nutrient availability, metabolites, and accompanying bacteria lead to differential ingestion of algae and associated microbes in eastern oysters (Newell and Jordan, 1983; Baldwin, 1995; Pales Espinosa et al., 2009; Nevejan et al., 2016). Interestingly, strong temporal changes were seen in the structure of microbial communities of oyster larvae, tank surface biofilms, and/or rearing water in each of the trials. Considering the short duration of the trials (less than 15 days), this indicates that temporal changes in bacterial communities in the tanks may be driven by developmental and health changes in the oyster larvae, since it is unlikely that these major changes are due to transient changes in the microbial composition of incoming water (as observed in Trial 3). More research is needed to evaluate the role of oyster-microbial interactions on the dynamics of microbial communities in rearing tanks in hatcheries.

There was no effect of probiotics on bacterial community diversity or structure in any of the sample types, suggesting that the primary probiotic effect of *B. pumilus* RI06-95 is exerted directly on larval health (e.g., by modulation of the immune system) and/or that it is mediated by subtle, targeted changes in the oyster microbiomes that are obscured by larger temporal effects and/or by homogenization of large pools of larvae from each tank. The presence of the probiotic was confirmed with higher relative abundance of *Bacillales* in the probiotic-treated water and increased relative abundance throughout the duration of each trial, suggesting that the probiotic accumulates in the larvae through time (tanks were scrubbed and water was changed every other day). Previous studies of the impact of probiotics on microbiota in humans and fish also showed subtle changes of certain taxa, but no consistent effect on the diversity of the host’s bacterial community (Boutin et al., 2013; Merrifield and Carnevali, 2014; Standen et al., 2015; Laursen et al., 2017; Schmidt et al., 2017). However, other studies report dramatic changes in fish intestinal microbiomes as a result of prebiotic treatment (Geraylou et al., 2013; Gonçalves and Gallardo-Escárate, 2017).

In addition to *Bacillales*, significant amplification of taxa was observed in probiotic-treated water samples compared to the control samples, most notably in the *Oceanospirillales* order. *Oceanospirillales* are heterotrophs commonly associated with mollusks and are found in the gills of many bivalves (Jensen et al., 2010; Zurel et al., 2011; Costa et al., 2012; Beinart et al., 2014). Additionally, they are recognized for their ability to degrade organic compounds in the environment and their abundance in oil plume microbial communities (Hazen et al., 2010; Dubinsky et al., 2013). These observations indicate that *Oceanospirillales* may confer a beneficial effect to the oyster host and contribute to the mechanism of oyster larval protection by the *B. pumilus* RI06-95 probiotic. Additionally, this suggests that the presence of *B. pumilus* RI06-95 has targeted effects on specific members of the microbial community in larval tanks in the hatchery.

Previous research showed that probiotic treatment with *B. pumilus* RI06-95 decreases levels of *Vibrio* spp. in the hatchery (Sohn et al., 2016a). This may be due to the production of antimicrobial secondary metabolites produced by *B. pumilus* RI06-95, as well as other *Bacillus* spp., that inhibit the growth of *Vibrio* spp. (Vaseeharan and Ramasamy, 2003; Karim et al., 2013; Sohn et al., 2016a). In the current study, a similar trend (as determined by a reduction in relative abundance, with overall trends confirmed using *Vibrio* spp. colony counts on selective media) was observed in treated tanks, but high variability and small sample sizes may have hindered detecting statistically significant differences. Moreover, failure to detect a significant decrease in *Vibrio* reads in Trial 2 (performed in January) was most probably due to the low abundance of *Vibrio* spp. in this trial, which is consistent with low levels of these species in coastal waters of the North Atlantic during winter (Staroscik and Smith, 2004). Interestingly, our research indicates that probiotic treatment leads to increased *Vibrio* diversity in rearing water through time. This increase in diversity in the absence of a net increase in relative abundance signifies a likely decrease in the relative abundance of specific pathogenic *Vibrio* spp., and therefore lower chances of a disease outbreak. Moreover, 16SrRNA oligotyping of the *Vibrio* species in the water samples revealed a transition in the *Vibrio* community in probiotic-treated tanks from a predominance of potentially pathogenic species [*V. alginolyticus*, a virulent pathogen originally isolated from amphioxus (Zou et al., 2016) and *V. celticus*, a virulent anaerobic clam pathogen (Beaz-Hidalgo et al., 2010b)] to a predominance of a likely non-pathogenic species [*V. orientalis*, a species that has been associated with adaptive functions (Tangl, 1983; Mukhta et al., 2016)]. This trend further confirms that addition of *B. pumilus* RI06-95 causes targeted changes in certain taxa, especially *Vibrios*, which is highly relevant for decreasing infective doses and, consequently, disease dynamics (Chauhan and Singh, 2018).

This interpretation is consistent with results from the co-occurrence network analysis, a tool used to identify associations, patterns, roles, and inform hypotheses from 16S abundance data (Barberán et al., 2012). This analysis suggests a negative association between *Bacillales* with *Vibrionales* in the trials performed in summer months (Trials 1 and 3), when *Vibrionales* are more abundant in the environment and oysters. Previous research and sequencing of the genome of *B. pumilus* RI06-95 show that potential mechanisms of probiotic action can include direct competition with other species and biofilm formation (Karim et al., 2013; Hamblin et al., 2015). Competition between *B. pumilus* RI06-95 and other bacteria (including *Vibrionales*) could open niches in the oyster microbiome for potentially beneficial microbes.

In summary, the bacterial community dynamics observed in this study indicate a variety of interactions between larval oysters and specific members of the microbiome, such as *Vibrio* spp. and the *Bacillus* probiotic. First, *Vibrio* spp., as well as other Proteobacteria, appear to be particularly capable of colonizing and surviving within oyster larvae (Romalde et al., 2014). These opportunistic *Vibrios* may be outcompeted by pre-colonization of other bacteria in the system, leading to a decrease in *Vibrio* abundance and/or an increase in diversity over time (Beaz-Hidalgo et al., 2010a; Zhao et al., 2016, 2018). We hypothesize that inhibition of *Vibrio* spp. by probiotic *B. pumilus* RI06-95 may allow for potentially beneficial *Oceanospirillales* to become more abundant in the system. Additional research is needed to examine the specific interactions between *Oceanospirillales* symbionts, the *Bacillus* probiotic, *Vibrio* pathogens, and the oyster host. Elucidating such interactions will require more targeted 16S rRNA and functional metagenomic analyses to track specific species over time, as well as functional studies using *in vitro* and *in vivo* competition experiments.

## Conclusion

This study investigated the effects of time and probiotic treatment on bacterial communities in an oyster hatchery. Understanding how probiotic treatment affects microbiota in aquaculture systems may help in optimizing their benefits and preventing undesirable side-effects (Kesarcodi-Watson et al., 2008). Our results show that there is a strong effect of time on the microbiomes within oyster larvae, on tank walls and in the rearing water, and that probiotic treatment leads to subtle changes in certain bacterial taxa, including an increase in the relative abundance of *Oceanospirillales* in the rearing water and changes in the *Vibrio* community. These results inform how probiotics may influence bacterial communities in an oyster hatchery over temporal and spatial scales, leading to an overall improvement in larval health.

## Data Availability

The raw sequences generated for this study can be found in the NCBI Short Read Archive under BioProject no. PRJNA518081. All processed 16S rRNA amplicon percent abundances at the Phylum and Order level and the R-markdown file to reproduce the figures in the manuscript can be found on Zenodo (https://doi.org/10.5281/zenodo.2658685). In addition, further public analysis and exploration of Trial 3 data are possible on the VAMPS website (https://vamps.mbl.edu/) using software under the project name AFP_RWU1_Bv6.

## Author Contributions

RJS, SS, DRN, DCR, AFP, and MG-C contributed conception and design of the study. SS, THM, KT, RS, and KML contributed to performance of the hatchery trials. RJS and SS collected and prepared the samples for sequencing. RJS performed the sequence analysis and wrote the manuscript. All authors contributed to manuscript revision, read and approved the submitted version.

## Conflict of Interest Statement

The authors declare that the research was conducted in the absence of any commercial or financial relationships that could be construed as a potential conflict of interest.

## Footnotes

1 https://vamps.mbl.edu/resources/primers.php
