# Supplementary material for: Bacterial Community Dynamics in an Oyster Hatchery in Response to Probiotic Treatment
Source: Front Microbiol. 2019 May 15;10:1060. doi: 10.3389/fmicb.2019.01060 (PMC6530434; doi:10.3389/fmicb.2019.01060)
Supplement: Supplementary file 1 [file Data_Sheet_1.PDF]

Supplementary Material

1 Supplementary Figures

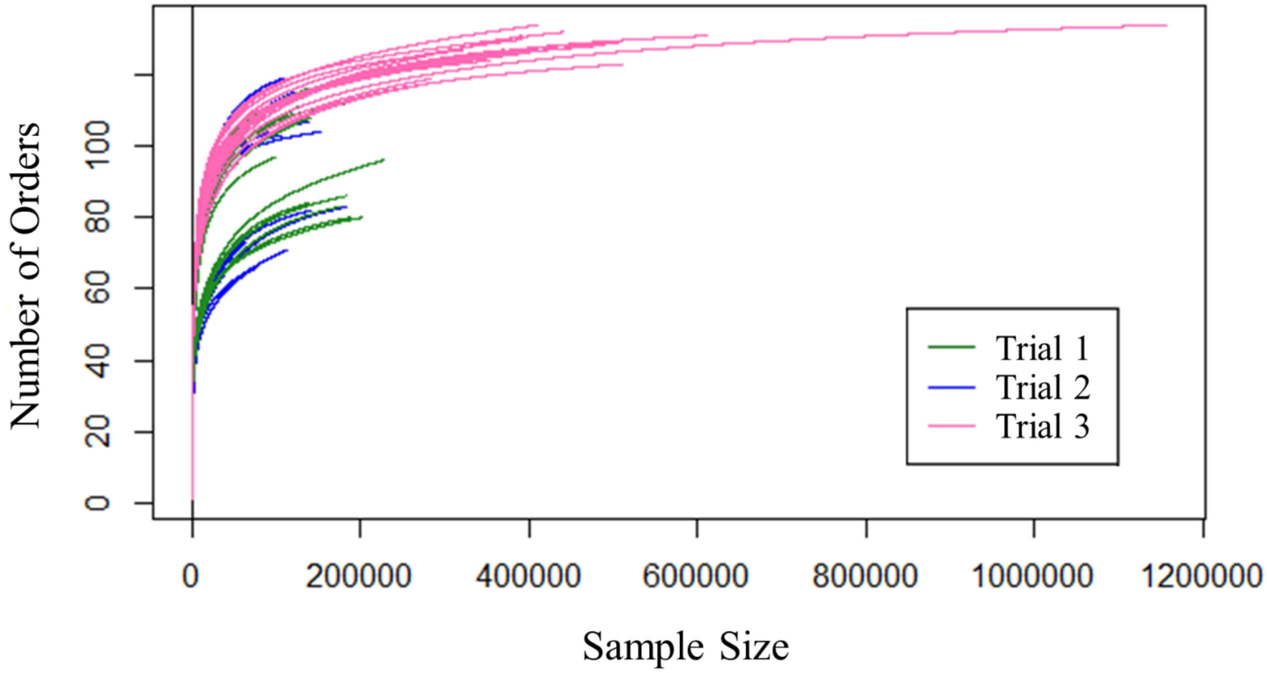

Supplementary Figure 1. Rarefaction curve from all water samples from all three Trials based on taxonomic classification at the order level.

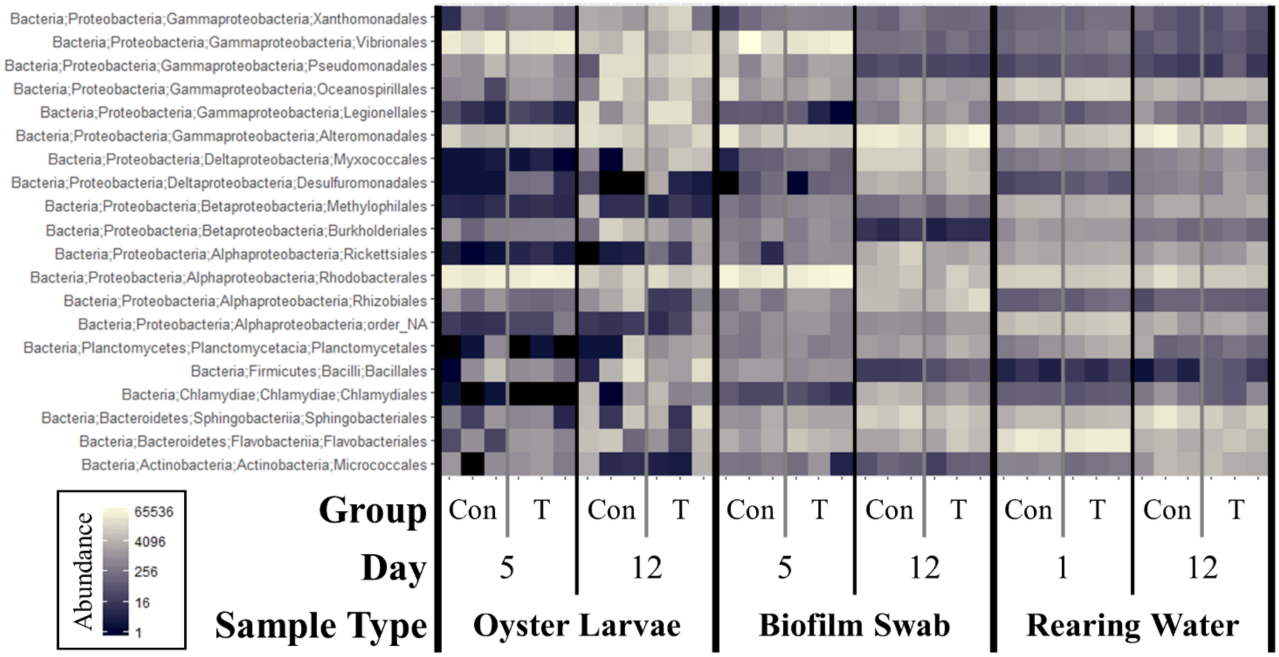

Supplementary Figure 2. The relative abundances of the 20 most abundant orders in oyster, swab, and water samples from Trial 1.

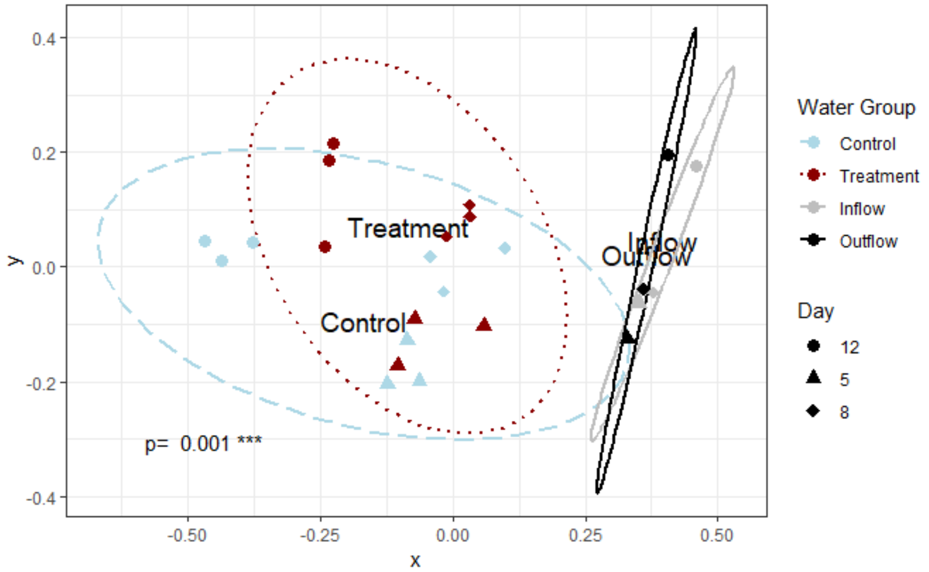

Supplementary Figure 3. NMDS plot visualization of Bray-Curtis beta-diversity (k=2) at the Order level by Treatment or Water Source of water samples from Trial 3. The ellipse lines show the 95% confidence interval. The water group is indicated by colors (control=light blue dashed, probiotic treatment=dark red dotted, inflow=grey solid, outflow=black solid) and sampling timepoints are indicated by symbols. The inflow water (water piped directly from the environment into the hatchery) and outflow water (inflow water UV-treated and sterilized) are significantly distinct groups, separate from the experimental samples. p-value indicates significance of groupings with adonis2 Permutational Multivariate Analysis of Variance Using Distance Matrices test.

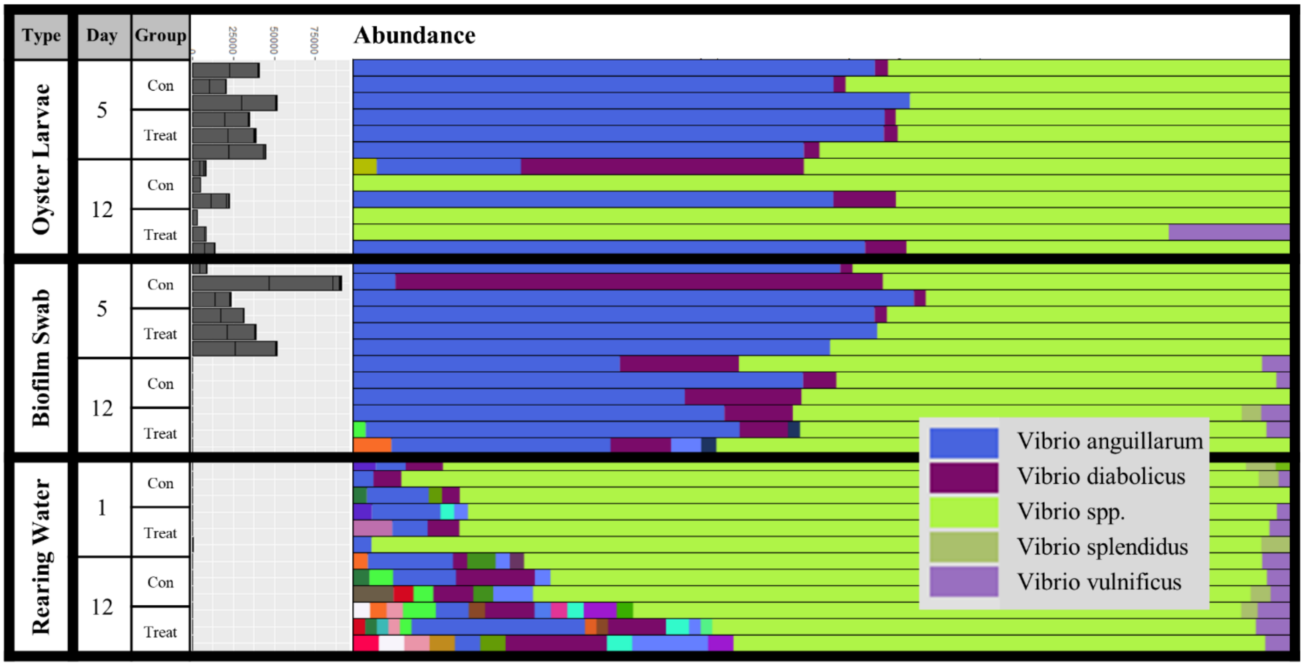

Supplementary Figure 4. Percent abundances of *Vibrio* species in all sample types in Trial 1. The total abundance of sequencing reads is shown in the bar graph. The structure of total *Vibrios* is different based on the sample type and time point.

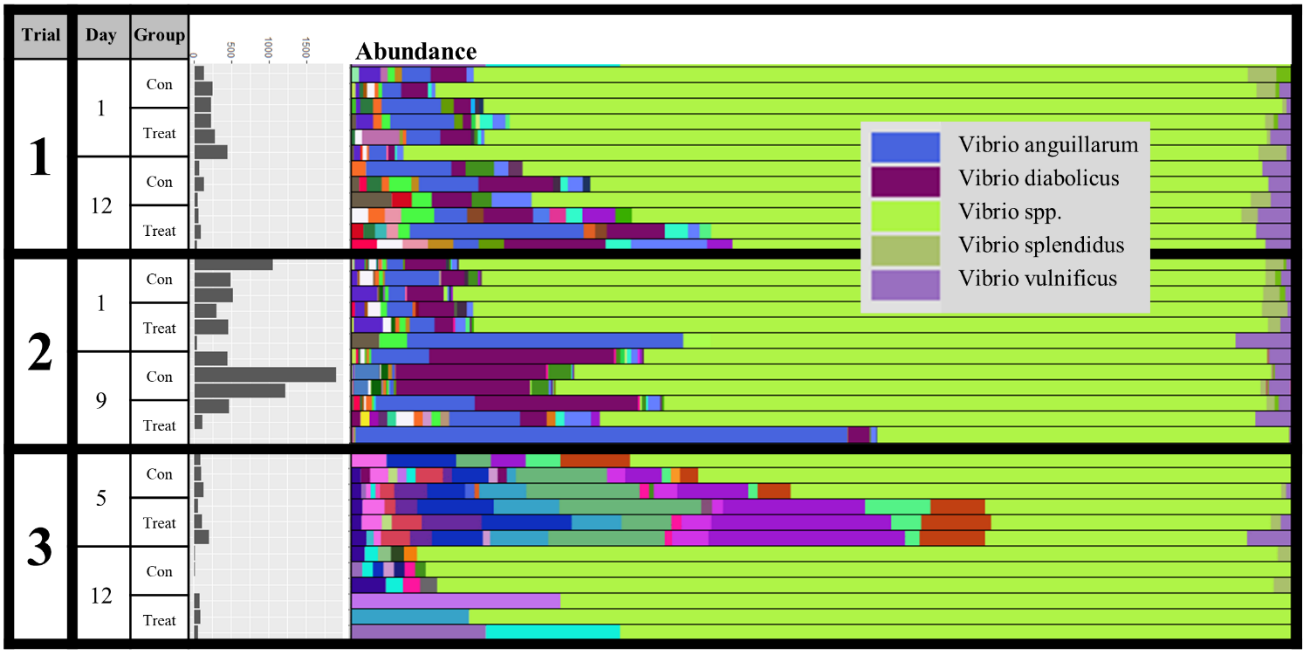

Supplementary Figure 5. Percent abundances of *Vibrio* species in rearing water samples from all 3 Trials. The total abundance of sequencing reads is shown in the bar graph. The structure of total *Vibrio* counts in the rearing water is different between Trials and changes over time.

2 Supplementary Tables

Supplementary Table 1. Kruskal-Wallis Rank Sum Test for percent abundances of *Proteobacteria*, *Cyanobacteria*, and *Bacteroidetes* by Sample Type from all trials.

**<All Trials – *Proteobacteria* >**				
	**DF**	**Chi-Squared**	**P value**	
Sample Type	2	52.745	3.521e-12	***
Treatment	1	0.75065	0.3863	
**< All Trials – *Cyanobacteria* >**				
	**DF**	**Chi-Squared**	**P value**	
Sample Type	2	33.113	6.451e-08	***
Treatment	1	0.093506	0.7598	
**< All Trials – *Bacteroidetes* >**				
	**DF**	**Chi-Squared**	**P value**	
Sample Type	2	63.422	1.691e-14	***
Treatment	1	0.23442	0.6283	

Supplementary Table 2. Kruskal-Wallis Rank Sum Test for the Simpson's Index of Diversity values by Trial, Sample Type, Day, and/or Treatment.

**< All Trials – Simpson's Index of Diversity >**				
	**DF**	**Chi-Squared**	**P value**	
Trial	2	38.553	4.25e-09	***
Trial – water only	2	24.809	4.099e-06	***
Type	2	51.932	5.285e-12	***
Day	2	9.1136	0.0105	*
Treatment	1	0.32388	0.5693	

Supplementary Table 3. Kruskal-Wallis Rank Sum Test for the Simpson's Index of Diversity values by Day and Treatment in Trial 1.

**< Trial 1 - Water >**				
	**DF**	**Chi-Squared**	**P value**	
Day	1	0.41026	0.5218	
Treatment	1	0.92308	0.3367	
**< Trial 1 – Biofilm Swab >**				
	**DF**	**Chi-Squared**	**P value**	
Day	1	8.3077	0.003948	**
Treatment	1	0.10256	0.7488	
**< Trial 1 – Oyster Larvae >**				
	**DF**	**Chi-Squared**	**P value**	
Day	1	6.5641	0.01041	*
Treatment	1	0.41026	0.5218	

Supplementary Table 4. Kruskal-Wallis Rank Sum Test for the Simpson's Index of Diversity values by Day and Treatment in Trial 2.

**< Trial 2 - Water >**				
	**DF**	**Chi-Squared**	**P value**	
Day	1	7.4103	0.006485	**
Treatment	1	0.10256	0.7488	
**< Trial 2 – Biofilm Swab >**				
	**DF**	**Chi-Squared**	**P value**	
Day	1	3.6923	0.05466	
Treatment	1	0.10256	0.7488	
**< Trial 2 – Oyster Larvae >**				
	**DF**	**Chi-Squared**	**P value**	
Day	1	0	1	
Treatment	1	0.6	0.4386	

Supplementary Table 5. Kruskal-Wallis Rank Sum Test for the Simpson's Index of Diversity values by Day and Treatment in Trial 3.

**< Trial 3 - Water >**				
	**DF**	**Chi-Squared**	**P value**	
Day	2	11.942	0.002552	**
Treatment	1	0.32943	0.566	

Supplementary Table 6. Permutational Multivariate Analysis of Variance Using Distance Matrices (adonis2) for Bray-Curtis beta-diversity (k=2) in each Trial by Sample Type, Day, and Treatment.

**< Trial 1 – Bray-Curtis beta-diversity >**						
	**DF**	**Sum of Squares**	**R^2**	**F**	**Pr(>F)**	
Type	2	2.0175	0.3053	7.2513	0.001	***
Residual	33	4.5907	0.6947			
Day (Water only)	1	0.71911	0.7375	28.095	0.006	**
Residual	10	0.25596	0.2625			
Treatment (Water only)	1	0.02426	0.02488	0.2551	0.719	
Residual	10	0.9508	0.97512			
**< Trial 2 – Bray-Curtis beta-diversity >**						
	**DF**	**Sum of Squares**	**R^2**	**F**	**Pr(>F)**	
Type	2	2.7762	0.57915	20.642	0.001	***
Residual	30	2.0173	0.42085			
Day (Water only)	1	0.18331	0.24214	3.195	0.013	*
Residual	10	0.57375	0.75786			
Treatment (Water only)	1	0.07318	0.09666	1.07	0.316	
Residual	10	0.68389	0.90334			
**< Trial 3 – Bray-Curtis beta-diversity >**						
	**DF**	**Sum of Squares**	**R^2**	**F**	**Pr(>F)**	
Day	1	0.14732	0.32893	7.8424	0.002	**
Residual	16	0.30056	0.67107			
Treatment	1	0.02889	0.06451	1.1033	0.337	
Residual	16	0.41898	0.93549			
Water Source Group (Figure S3)	3	0.37087	0.57173	8.9	0.001	***
Residual	20	0.27781	0.42827			

Supplementary Table 7. Kruskal-Wallis Rank Sum Test for relative percent abundance of *Bacillales* reads in water samples per Trial by Day and Treatment Group.

**< Trial 1 - *Bacillales* >**				
	**DF**	**Chi-Squared**	**P value**	
Day	1	0.64103	0.4233	
Treatment: Day 1	1	1.1905	0.2752	
Treatment: Day 12	1	3.8571	0.04953	*
**< Trial 2 - *Bacillales* >**				
	**DF**	**Chi-Squared**	**P value**	
Day	1	0.23077	0.631	
Treatment: Day 1	1	0.42857	0.5127	
Treatment: Day 9	1	3.8571	0.04953	*
**< Trial 3 - *Bacillales* >**				
	**DF**	**Chi-Squared**	**P value**	
Day	2	0.94737	0.6227	
Treatment: All	1	12.789	0.0003486	***
Treatment: Day 5	1	3.8571	0.04953	*
Treatment: Day 8	1	3.8571	0.04953	*
Treatment: Day 12	1	3.8571	0.04953	*
**< Trial 3 - *Bacillales* Days 5 and 8 >**				
	**DF**	**Chi-Squared**	**P value**	
Day	1	3.8571	0.04953	*
**< Trial 3 - *Bacillales* Days 5 and 12 >**				
	**DF**	**Chi-Squared**	**P value**	
Day	1	3.8571	0.04953	*
**< Trial 3 - *Bacillales* Days 8 and 12 >**				
	**DF**	**Chi-Squared**	**P value**	
Day	1	3.8571	0.04953	*

Supplementary Table 8. Kruskal-Wallis Rank Sum Test for relative percent abundance of *Oceanospirillales* reads in water samples per Trial by Day and Treatment Group.

**< Trial 1 - *Oceanospirillales* >**				
	**DF**	**Chi-Squared**	**P value**	
Day	1	8.3077	0.003948	**
Treatment: Day 1	1	3.8571	0.04953	*
Treatment: Day 12	1	3.8571	0.04953	*
**< Trial 2 - *Oceanospirillales* >**				
	**DF**	**Chi-Squared**	**P value**	
Day	1	5.7692	0.01631	*
Treatment: Day 1	1	3.8571	0.04953	*
Treatment: Day 9	1	1.1905	0.2752	
**< Trial 3 - *Oceanospirillales* >**				
	**DF**	**Chi-Squared**	**P value**	
Day	2	8.2222	0.01639	*
Treatment: Day 5	1	3.8571	0.04953	*
Treatment: Day 8	1	3.8571	0.04953	*
Treatment: Day 12	1	3.8571	0.04953	*
**< Trial 3 - *Oceanospirillales* Days 5 and 8 >**				
	**DF**	**Chi-Squared**	**P value**	
Day	1	4.3333	0.03737	*
**< Trial 3 - *Oceanospirillales* Days 5 and 12 >**				
	**DF**	**Chi-Squared**	**P value**	
Day	1	1.2564	0.2623	
**< Trial 3 - *Oceanospirillales* Days 8 and 12 >**				
	**DF**	**Chi-Squared**	**P value**	
Day	1	6.5641	0.01041	*

Supplementary Table 9. Kruskal-Wallis Rank Sum Test for Simpson's Index of Diversity of *Vibrionales* relative percent reads in Trial 1 per Sample Type by Day and Treatment Group.

**< Trial 1 All Samples – *Vibrio* diversity >**				
	**DF**	**Chi-Squared**	**P value**	
Type	2	8.4324	0.01475	*
Day	2	10.89	0.004318	**
Treatment	1	0.25626	0.6127	
**< Trial 1 Oyster Larvae – *Vibrio* diversity >**				
	**DF**	**Chi-Squared**	**P value**	
Day	1	0	1	
Treatment	1	0.41026	0.5218	
**< Trial 1 Biofilm swab – *Vibrio* diversity >**				
	**DF**	**Chi-Squared**	**P value**	
Day	1	8.3077	0.003948	**
Treatment	1	0.025641	0.8728	
**< Trial 1 Water – *Vibrio* diversity >**				
	**DF**	**Chi-Squared**	**P value**	
Day	1	8.3077	0.003948	**
Treatment	1	0.64193	0.4233	
Treatment – Day 12	1	3.8571	0.04953	*

Supplementary Table 10. Kruskal-Wallis Rank Sum Test for relative percent abundance of *Vibrionales* reads in Trial 1 per Sample Type by Day and Treatment Group.

**< Trial 1 All Samples – *Vibrio* percent abundance >**				
	**DF**	**Chi-Squared**	**P value**	
Type	2	16.722	0.0002338	***
Day	2	22.651	1.206e-05	***
Treatment	1	0.0009009	0.9244	
**< Trial 1 Oyster Larvae – *Vibrio* percent abundance >**				
	**DF**	**Chi-Squared**	**P value**	
Day	1	8.3077	0.003948	**
Treatment	1	0.10256	0.7488	
**< Trial 1 Biofilm swab – *Vibrio* percent abundance >**				
	**DF**	**Chi-Squared**	**P value**	
Day	1	8.3077	0.003948	**
Treatment	1	0	1	
**< Trial 1 Water – *Vibrio* percent abundance >**				
	**DF**	**Chi-Squared**	**P value**	
Day	1	8.3077	0.003948	**
Treatment	1	0.025641	0.8728	

Supplementary Table 11. Kruskal-Wallis Rank Sum Test for culturable *Vibrio* colony counts in Trial 1 per Sample Type by Day and Treatment Group.

**< Trial 1 All Samples – *Vibrio* colony counts >**				
	**DF**	**Chi-Squared**	**P value**	
Type	2	2.4254	0.2974	
Day	2	2.4406	0.2951	
Treatment	1	10.234	0.001379	**
**< Trial 1 Oyster Larvae – *Vibrio* colony counts >**				
	**DF**	**Chi-Squared**	**P value**	
Day	1	3.7053	0.05424	
Treatment	1	1.8591	0.1727	
**< Trial 1 Biofilm swab – *Vibrio* colony counts >**				
	**DF**	**Chi-Squared**	**P value**	
Day	1	2.0769	0.1495	
Treatment	1	3.1026	0.07817	
**< Trial 1 Water – *Vibrio* colony counts >**				
	**DF**	**Chi-Squared**	**P value**	
Day	1	2.0989	0.1474	
Control by Day	1	3.9706	0.0463	*
Treated by Day	1	4.0909	0.04311	*
Treatment	1	8.3958	0.003761	**
